# 1935. COVID-19 mRNA Vaccination Reduces the Occurrence of Post-COVID Conditions in U.S. Children Aged 5-17 Years Following Omicron SARS-CoV-2 Infection, July 2021-September 2022

**DOI:** 10.1093/ofid/ofad500.2466

**Published:** 2023-11-27

**Authors:** Anna R Yousaf, Josephine Mak, Lisa Gwynn, Robin Bloodworth, Ramona Rai, Zuha Jeddy, Lindsay B LeClair, Laura Edwards, Lauren E W Olsho, Gabriella Newes-Adeyi, Alexandra F Dalton, Manjusha Gaglani, Sarang K Yoon, Kurt Hegmann, Katherine Ellingson, Leora R Feldstein, Angela P Campbell, Amadea Britton, Sharon Saydah

**Affiliations:** Centers for Disease Control and Prevention, Atlanta, GA; Division of Healthcare Quality Promotion, Centers for Disease Control and Prevention, Atlanta, Georgia; Miller School of Medicine, University of Miami, Miami, Florida; Abt Associates, Rockville, Maryland; Abt Associates, Rockville, Maryland; Abt Associates, Rockville, Maryland; Abt Associates, Rockville, Maryland; Abt Associates, Rockville, Maryland; Abt Associates, Rockville, Maryland; Abt Associates, Rockville, Maryland; Centers for Disease Control and Prevention, Atlanta, GA; Baylor Scott & White Health, Temple, TX; University of Utah School of Medicine, Salt Lake City, Utah; U.Utah, SLC, Utah; University of Arizona, Tucson, Arizona; Centers for Disease Control and Prevention, Atlanta, GA; Centers for Disease Control and Prevention, Atlanta, GA; Centers for Disease Control and Prevention, Atlanta, GA; Centers for Disease Control and Prevention, Atlanta, GA

## Abstract

**Background:**

An estimated 1-3% of children with SARS-CoV-2 infection will develop Post-COVID Conditions (PCC). This study evaluates mRNA COVID-19 vaccine impact on likelihood of PCC in children.

**Methods:**

A multi-site cohort of children enrolled 7/21/2021-9/1/2022 underwent weekly SARS-CoV-2 screening tests and were surveyed via self- or parental report 12/1/2022-5/31/2023 regarding PCC (defined as ≥1 new or on-going symptoms lasting ≥ 1 month after infection). Multivariable logistic regression was performed to estimate the occurrence of PCC by vaccination status among children aged 5–17 years whose first PCR-confirmed SARS-CoV-2 infection occurred in-study with Omicron variant, who completed the survey >60 days from infection, and who were vaccine age-eligible at time of infection per ACIP recommendations. Vaccination status was categorized as vaccinated (at least primary series completed >14 days before infection) and unvaccinated (no vaccine doses before infection). Vaccination status was verified through vaccine registry and/or medical records.

**Results:**

Of 622 participants surveyed, 5% (n=28) had PCC (Table 1) and 67% (n=474) were vaccinated (Table 2). Surveys were completed a median (IQR) of 203.7 days (119.0–293.0) after infection. Children with non-Hispanic Black race/ethnicity and good/fair/poor self-rated baseline health were more likely to report PCC. Children aged 12-18 years, Non-Hispanic Asian and White children, those reporting symptomatic SARS-CoV-2 infection, and those with excellent/very good self-rated baseline health were more likely to report vaccination When comparing children with and without PCC symptoms, COVID-19 mRNA vaccination was associated with a decreased likelihood of >1 PCC symptom (aOR 0.66, 95% CI 0.43-0.99), >2 PCC symptoms (aOR 0.52, 95% 0.32-0.83), and respiratory PCC symptoms (aOR 0.53, 95% CI 0.33-0.87) (Table 3).

**Table 1.** Characteristics of all study participants by report of Post-COVID Conditions (PCC) on survey, N=622.

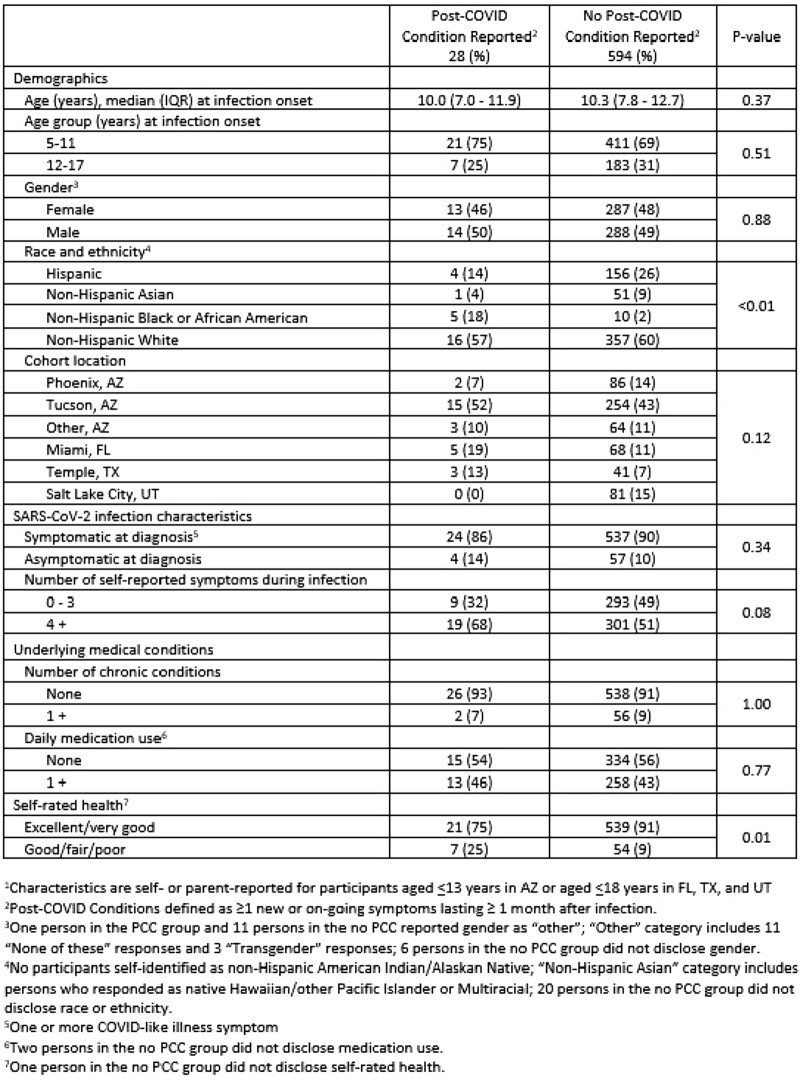

**Table 2.** Characteristics of all study participants by COVID-19 vaccination status, N=622.

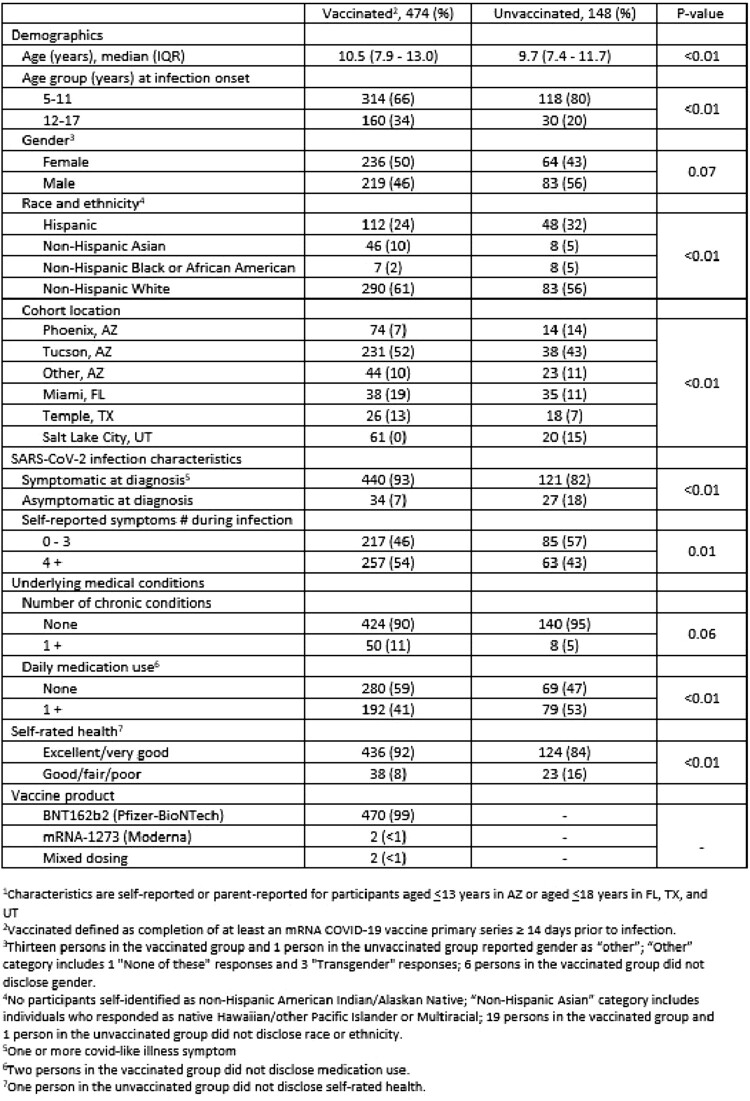

**Table 3.** Adjusted odds of Post-COVID conditions (PCC) by COVID-19 vaccination status.

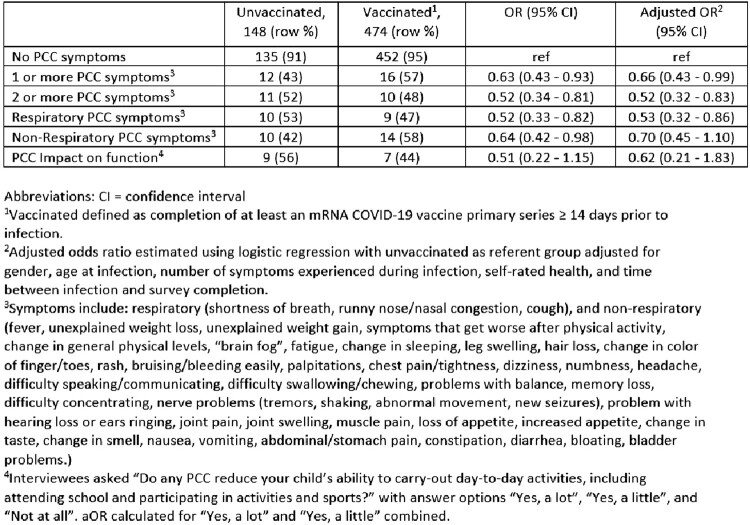

**Conclusion:**

In this study, mRNA COVID-19 vaccination appeared to be protective against PCC in children following Omicron SARS-CoV-2 infection. The adjusted ORs correspond to an estimated 34%, 48%, and 47% reduced likelihood of >1, >2, and respiratory PCC symptoms among vaccinated children, respectively. These findings support COVID-19 vaccination for children and may encourage increased pediatric vaccine uptake.

**Disclosures:**

**Lisa Gwynn, MBA, MSPH**, Merck: Honoraria

