# Correction to: 1935. COVID-19 mRNA Vaccination Reduces the Occurrence of Post-COVID Conditions in U.S. Children Aged 5-17 Years Following Omicron SARS-CoV-2 Infection, July 2021-September 2022

**DOI:** 10.1093/ofid/ofae100

**Published:** 2024-02-20

In the originally published version of this abstract, [Yousaf AR, Mak J, Gwynn L et al. 1935. COVID-19 mRNA Vaccination Reduces the Occurrence of Post-COVID Conditions in U.S. Children Aged 5-17 Years Following Omicron SARS-CoV-2 Infection, July 2021-September 2022. Open Forum Infectious Diseases, Volume 10, Issue Supplement_2, December 2023, ofad500.2466, https://doi.org/10.1093/ofid/ofad500.2466] there were three figures from another abstract in this supplement that were erroneously published in place of the three accepted tables. The missing tables have been reinserted into the version of this abstract currently available online.

